# “Is It Removed During Dialysis?”—Cognitive Dysfunction in Advanced Kidney Failure—A Review Article

**DOI:** 10.3389/fneur.2021.787370

**Published:** 2021-12-02

**Authors:** Kirsty Crowe, Terence J. Quinn, Patrick B. Mark, Mark D. Findlay

**Affiliations:** ^1^Glasgow Renal and Transplant Unit, Queen Elizabeth University Hospital, Glasgow, United Kingdom; ^2^Institute of Cardiovascular and Medical Sciences, University of Glasgow, Glasgow, United Kingdom

**Keywords:** cognitive dysfunction, kidney failure, neurocognitive disorder, dialysis, dementia, cognitive impairment, uremia

## Abstract

Cognitive impairment is independently associated with kidney disease and increases in prevalence with declining kidney function. At the stage where kidney replacement therapy is required, with dialysis or transplantation, cognitive impairment is up to three times more common, and can present at a younger age. This is not a new phenomenon. The cognitive interactions of kidney disease are long recognized from historical accounts of uremic encephalopathy and so-called “dialysis dementia” to the more recent recognition of cognitive impairment in those undergoing kidney replacement therapy (KRT). The understanding of cognitive impairment as an extra-renal complication of kidney failure and effect of its treatments is a rapidly developing area of renal medicine. Multiple proposed mechanisms contribute to this burden. Advanced vascular aging, significant multi-morbidity, mood disorders, and sleep dysregulation are common in addition to the disease-specific effects of uremic toxins, chronic inflammation, and the effect of dialysis itself. The impact of cognitive impairment on people living with kidney disease is vast ranging from increased hospitalization and mortality to decreased quality of life and altered decision making. Assessment of cognition in patients attending for renal care could have benefits. However, in the context of a busy clinical service, a pragmatic approach to assessing cognitive function is necessary and requires consideration of the purpose of testing and resources available. Limited evidence exists to support treatments to mitigate the degree of cognitive impairment observed, but promising interventions include physical or cognitive exercise, alteration to the dialysis treatment and kidney transplantation. In this review we present the history of cognitive impairment in those with kidney failure, and the current understanding of the mechanisms, effects, and implications of impaired cognition. We provide a practical approach to clinical assessment and discuss evidence-supported treatments and future directions in this ever-expanding area which is pivotal to our patients' quality and quantity of life.

## Introduction

Cognitive impairment is increasingly prevalent in aging and multi-morbid populations. Chronic kidney disease (CKD) is also increasing in prevalence and after correcting for shared vascular risk factors within an aging multi-morbid population, advanced CKD is independently associated with cognitive impairment (1). Kidney replacement therapies (KRT) with dialysis or kidney transplantation carry a cognitive burden in themselves, from the lifestyle demands and healthcare interactions demands on patients through to marked physiological stressors and unique cardiovascular instability. The reciprocal relationship between cognitive impairment and CKD has been recognized for a long time, although a full understanding of this relationship and how to address it has only relatively recently become the focus of research activity.

In this article we provide an overview of the relationship of cognitive impairment and kidney failure—examining the past, present and looking to the future. After reflecting on the historical context of cognitive impairment in kidney failure, current epidemiological patterns, and presentation of cognitive impairment in people with kidney failure with replacement therapy (KFRT) will be discussed. The proposed mechanisms underlying the relationship between cognitive impairment and KFRT and the real-life patient implications of cognitive impairment will be considered. Methods of assessing cognitive impairment in the KFRT population and possible treatment and preventative strategies will be examined, followed by a discussion of future areas for research.

## “Has Long Been Known...”—The History of Cognitive Impairment in Kidney Failure

Nephrology as we know it now—a multifaceted specialty comprising immunology, dialysis, transplantation and hypertension—is a relatively young specialty. To put this in context, coinage of the word Nephrology and its recognition as a unique specialty is often attributed to the “Premier Congrès International de Néphrologie”—the first meeting of the International Society of Nephrology (2), held in 1960. Early a primary focus was, and remains, prevention and treatment of renal failure. In an aging and increasingly comorbid population the wider complications of kidney disease and its treatments are now claiming precedence (3).

**Figure 1:**
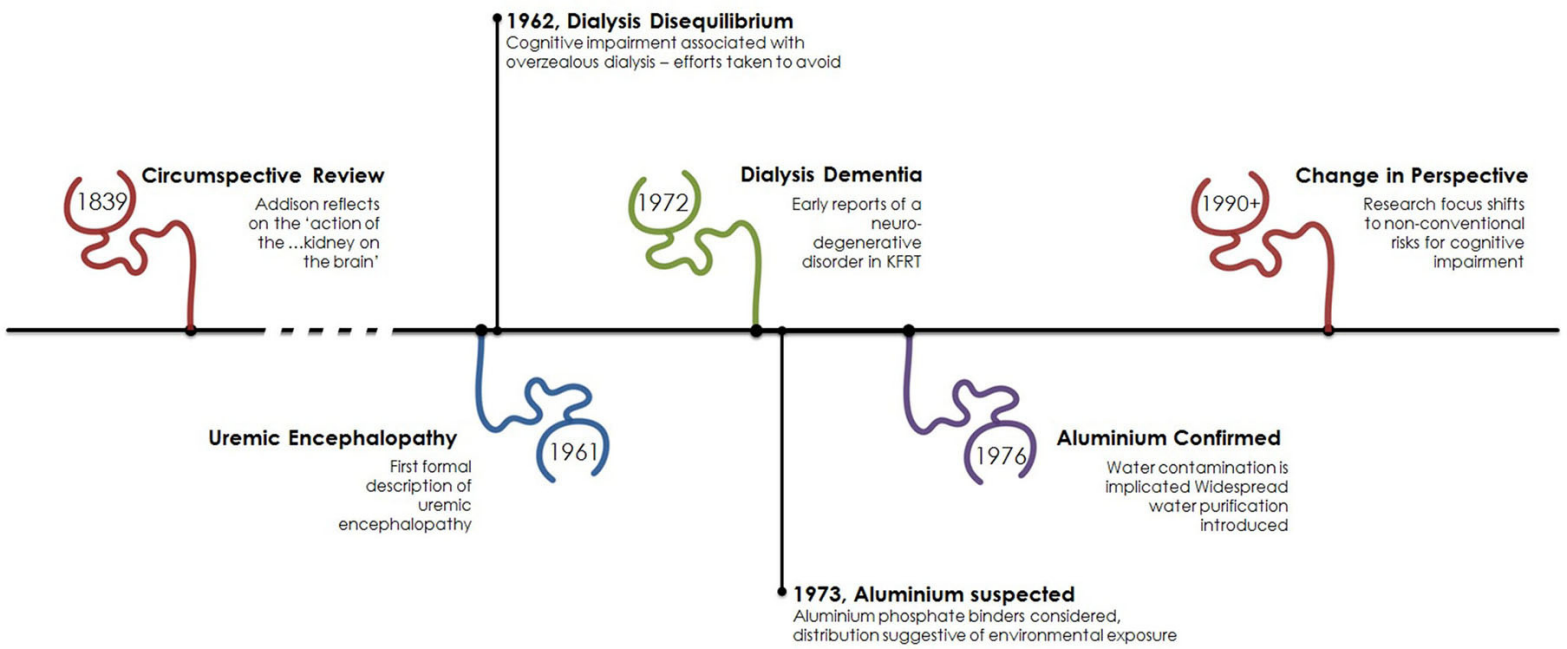
Timeline of cognitive impairment in kidney failure. The association of cognitive impairment and kidney disease has been recognized for some time with early focus on the neurotoxic effect of kidney failure. Following the advent of dialysis the process of dialysis was responsible for cerebral dysfunction as a result of its own associated initially unrecognized neurotoxin, aluminum. From the early 1990s focus has turned toward other factors associated with kidney failure such as anemia and new dialysis-specific effects.

However, it must be noted the effect of kidney disease on cognitive function is not a new observation as the “…*reciprocal action of the brain on the kidney and the kidney on the brain, has long been known*.” Written in 1839, in volume 31 of the Medico-Chirurgical review and Journal of Practice Medicine Dr. Thomas Addison describes this “new ground” in clinical observation (4). In his review he describes a variety of clinical presentations, including “…dullness of intellect...” and “…sluggishness of manner…” that affect those with renal disease. It seems likely Addison, an English physician and scientist, was describing uremic encephalopathy over a century before the birth of modern nephrology, see Figure 1.

Described in greater detail by Schreiner and Maher in 1961 (5), uremic encephalopathy is now fortunately a rarely witnessed complication of kidney failure at the point where dialysis is indicated. Presenting as lethargy, irritability, disorientation, hallucinations, and altered speech uremic encephalopathy can progress to tremor, myoclonus, seizures, and coma. Rarely, focal neurological signs such as hemiparesis can be present, and are unusually transient and can alternate from side to side (6). Although branded uremic encephalopathy and observation that the severity of symptoms parallels the degree of kidney dysfunction (and thus urea concentration), it remains unclear which toxin(s) are responsible for this clinical syndrome. Altered neurotransmitter function (7), acidosis (8), and elevated PTH (9) are amongst the suggested mechanisms. Diagnosis is clinical, and the continued treatment with dialysis resolves the majority of symptoms.

**Figure 2:**
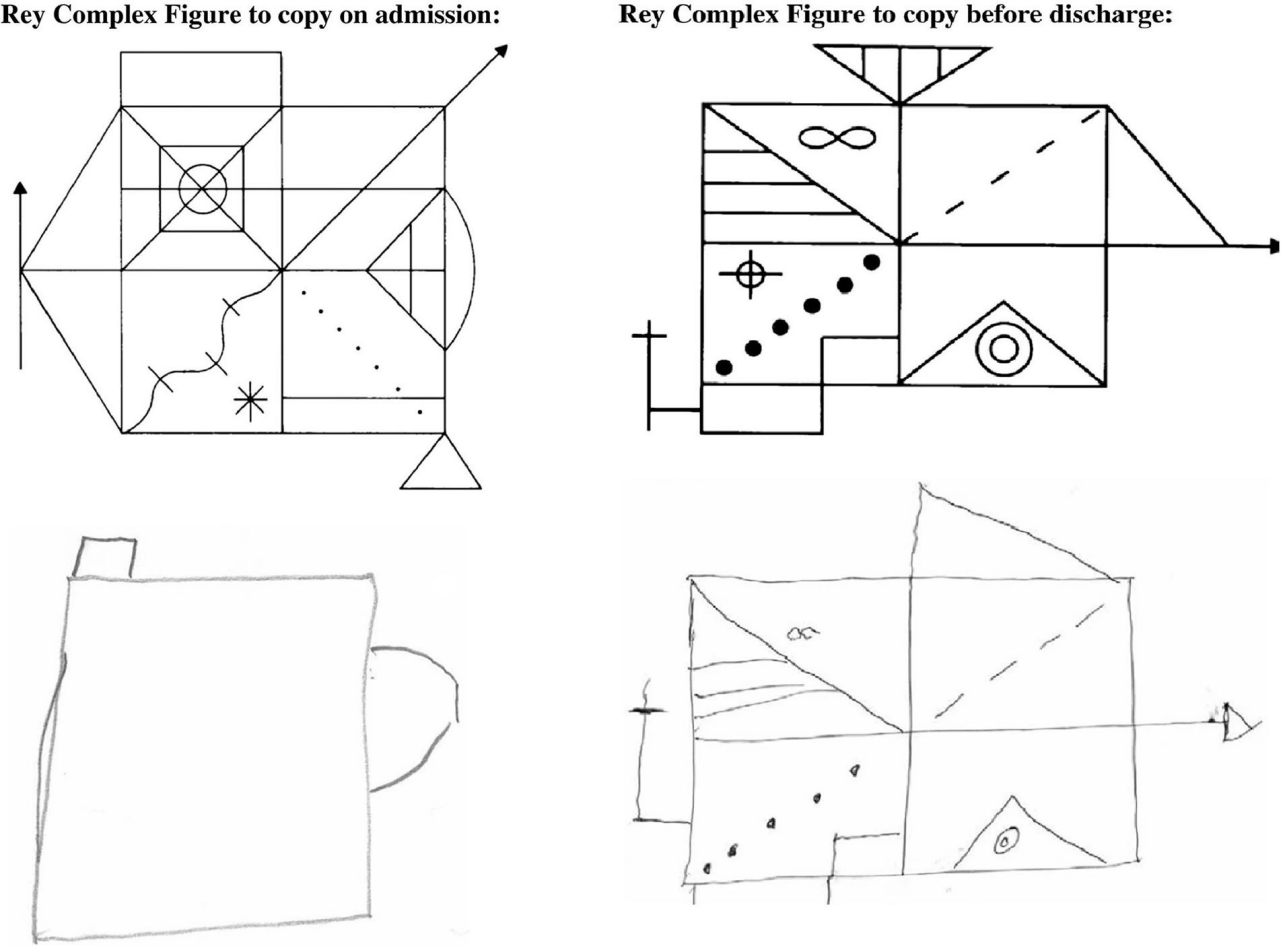
Rey Complex figure testing before and after correction of severe kidney failure. This assessment captures multiple domains of cognitive function including memory, processing speed, and visuospatial construction ability. In this example the admission assessment, performed with a serum creatinine of 2,443 umol/L and blood urea of 67.6 mmol/L is compared to the discharge assessment (creatinine 629 umol/L, urea 17.8 mmol/L). Reproduced from Schneider et al. (10) under the Creative Commons Attribution License.

Although acute kidney injury is capable of causing similar cerebral dysfunction, and often labeled delirium of acute illness, modern nephrologists rarely witness such marked neurocognitive impairment attributable to the uremic encephalopathy of advanced kidney failure. However, such an insight was described in a recent case report from 2012 where a 27-year-old presented with advanced kidney transplant failure—serum creatinine 2443 umol/L and urea 67.6 mmol/L. A detailed neurocognitive assessment was serendipitously available due to ongoing research and its use demonstrated sequential improvement in executive function and attention with subsequent dialysis treatment, Figure 2 (10).

Thus, with the advent of regular hemodialysis the neurotoxicity of kidney disease could now be alleviated as nephrologists provide respite. Describing the first two regular hemodialysis patients Scribner et al. highlight that sufficient dialysis led to improvement where “*neither patient has yet shown the relentless loss of weight and the mental deterioration which has been encountered in the past when less intensive dialysis therapy was employed”*(11). Dialysis was a success. For about a decade.

**Figure 3:**
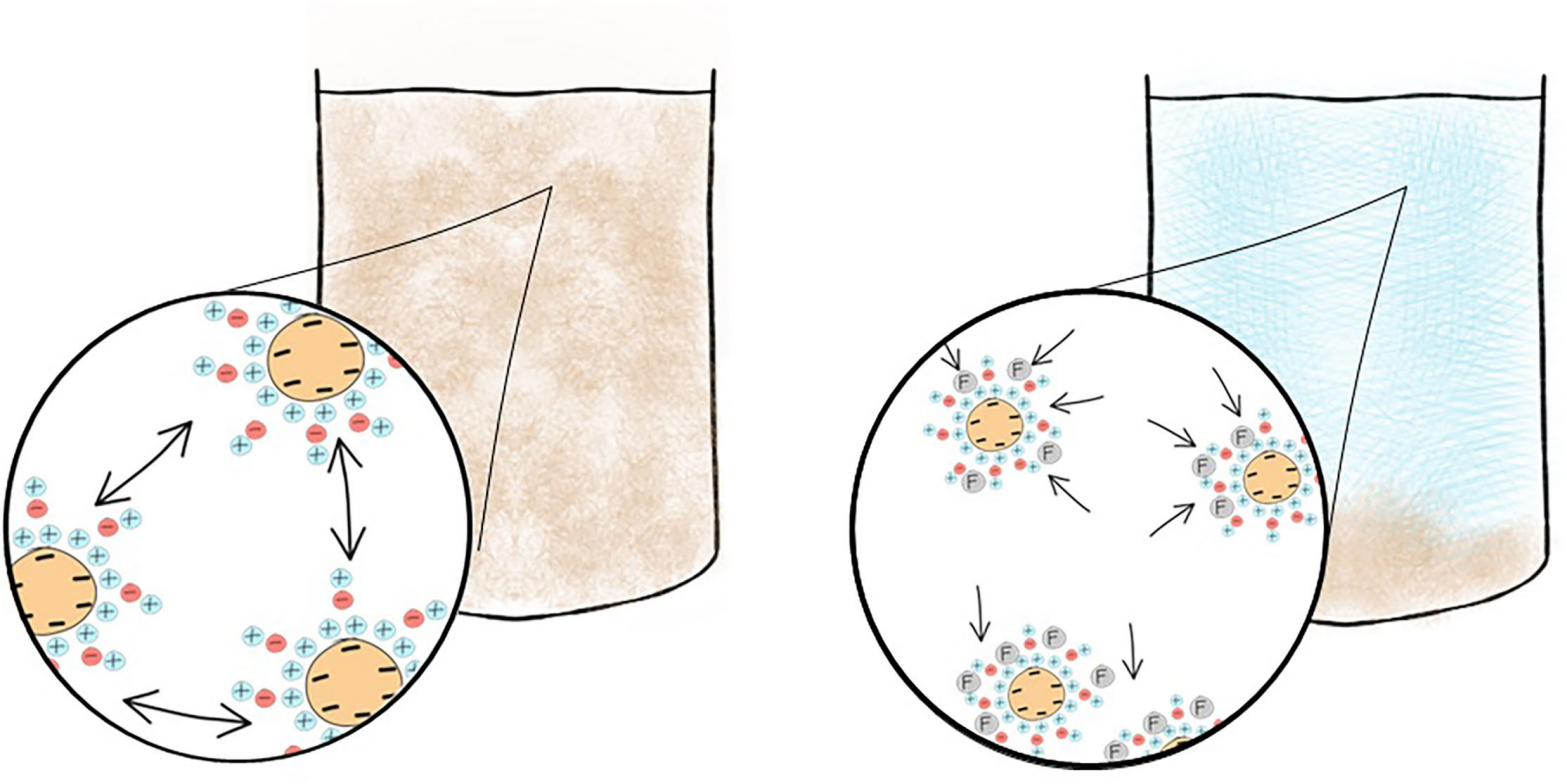
Addition of a flocculant such as aluminum improves water clarity prior to public consumption. Colloidal suspensions persist when the particulate matter is too light to sink and their surrounding charge prevents the colloids from adhering to each other. The addition of a flocculant overcomes this causing the colloid to form flocs which will sediment within the body of water. Aluminum, a flocculant, is added to drinking water and now removed by reverse osmosis prior to use in dialysis preventing toxicity.

First described in 1972 (12) a syndrome of dysarthria, dyspraxia and speech problems leading to personality problems, seizures, dementia and death was emerging in patients on dialysis. Aluminum, a heavy metal, was suspected to cause this neurodegenerative disorder and in 1970 high serum aluminum levels were discovered and initially attributed to the use of aluminum hydroxide used as a phosphate binder (13). Coined “dialysis dementia,” keen observation recognized an association with symptoms being “aggravated during and immediately following dialysis” (12) and geographical clustering led investigators to suspect an environmental factor. In 1976, a report from the Netherlands (14) described discrepancy of cases between two hemodialysis units. In the affected unit, high concentrations of aluminum were found in the dialysis water due to an eroded heating element. Evidence was growing to support aluminum in the water supply was culprit (15). However, the rare occurrence of faulty equipment did not explain the observed international outbreak. Two years later a report in the BMJ was published from a group in Glasgow, Scotland demonstrating a correlation between local prevalence of dialysis encephalopathy and aluminum concentration in tap water (16) used for dialysis. Routinely, aluminum is added to drinking water to promote flocculation, a process where suspended particles clump together to improve clarity of the water, Figure 3.

In areas with soft water, such as the west of Scotland—where the use of reverse osmosis purification was not felt necessary to reduce calcium content prior to use—dialysis patients would be exposed to high levels of aluminum with each session. Since its recognition and focus on improving dialysis through strict water standards “dialysis dementia” is a thing of the past.

As the process of dialysis has become safer and more efficient and the population receiving dialysis more medically complex attention has turned toward recognizing cognitive dysfunction as a comorbidity in those with kidney failure and exploring the mechanisms which are responsible for it.

## A Common but Poorly Recognized Problem—Epidemiology

Cognitive impairment is common in kidney failure with replacement therapy (KFRT) (17). In order to discuss cognitive impairment, it is essential to consider a few definitions. For the purposes of clinical assessment, cognitive function is conveniently divided into individual domains. Commonly assessed domains include attention, memory, language, visuospatial perception, social cognition and executive function, which is the ability to plan and carry out complex tasks. These are the domains that are used to assess for the clinical syndrome of dementia in the latest iteration of the Diagnostics and Statistics Manual (DSM-5). Assessment tools exist to determine deficits in one or multiple domains (discussed later) and are used to assess the possible etiology, severity and consequences of cognitive impairment. The commonly labeled “mild cognitive impairment” (MCI), or strictly “mild neurocognitive disorder” in the DSM-5, is defined as a “moderate cognitive decline from a previous level in ≥1 cognitive domain,” which is neither attributable to delirium nor another mental disorder and does not interfere with independence in daily activities. In contrast, dementia (or “major neurocognitive disorder”) is defined by presence of “major decline,” usually over two domains and which is sufficient to interfere with independence in everyday activities (18). Thus, it is the functional impact of the cognitive impairment that determines the eventual diagnostic formulation and emphasizes the importance of assessing not only cognitive domains but also how these impairments interfere with daily life.

Various factors influence the prevalence of cognitive impairment. Twelve modifiable risk factors which account for 40% of worldwide dementias include excessive alcohol use, smoking, physical inactivity, low social contact, diabetes, and hearing impairment (19). The single greatest factor in the general population is age (20, 21). Definitions of cognitive impairment and characteristics of included populations vary considerably between studies, and for this reason there is significant variation in reported prevalence. However, the authors of the COSMIC collaboration accumulated the results of 10 studies—collectively 20,987 participants—in an attempt to quantify this within a diverse geographical and ethno-cultural population. Using an MMSE score 24–27 they described a crude incidence of 12% in those with mean age range of 68.5–78.3 years (22).

So, how does this compare to the KFRT population? Executive function which encompasses higher cognitive processing such as planning, task prioritization and self-regulation, is the most commonly affected cognitive domain in kidney disease, reflecting the sub-cortical vascular impairment in this population (23, 24). There is a recognized linear relationship between GFR and prevalence of cognitive impairment. It is estimated that for every 10 ml/min/1.73m^2^ decrease in GFR in those aged >55 years there is an 11% increase in prevalence of cognitive impairment (25). Further, as the eGFR falls below 45 mL/min/1.73^2^ cognitive function declines substantially (26, 27), and cognitive decline accelerates more rapidly with eGFR below 30 mL/min/1.73^2^ (28). At the point of KFRT the estimated prevalence of cognitive impairment ranges from 27 to 77% (17, 29). Many patients with KFRT are excluded from completing standard cognitive assessments due to physical disability from previous vascular events which results in underestimation of prevalence by excluding those with an established vascular burden (30). Additionally many studies attempting to ascertain the prevalence of cognitive impairment in these populations have missing data due to poor patient motivation for completing assessments, which can itself be a consequence of cognitive impairment.

In 2018 in the UK the median age of those receiving in-center hemodialysis was 67.4 years, having risen from 63.3 years, since 2000 (31). Therefore, in an ever-aging population, with such high burden of cognitive impairment nephrologists may become overwhelmed with concerns previously thought to be those of geriatricians. In a US publication from 2006, it was estimated that over 80% of a dialysis cohort (*n* = 338), mean age 71.2 years had cognitive impairment. They described 13.9% as having mild, 36.1% moderate, and 37.3% severe cognitive impairment. Most striking, in this same cohort only 2.9% had an existing clinical label of cognitive impairment (17).

Recognition of cognitive impairment is poor but perhaps this relates to the insidious onset throughout CKD stages and a potential recovery following initiation of dialysis? This does not appear to be the case. The authors of the Chronic Renal Insufficiency Cohort (CRIC) study recognized that the initiation of dialysis can induce a stepwise decline in cognitive function—specifically executive function. In their study they prospectively followed over 200 patients with CKD likely to progress to hemodialysis, or who had progressed to hemodialysis, over a 2-year period. Three groups of patients emerged, those who remained in CKD, those who transitioned to dialysis and those who were on dialysis at first cognitive assessment. A significant drop in executive function was noted in those who transitioned to dialysis during follow-up, with progressive decline at a rate similar to those on dialysis. The executive function of those who did not require dialysis declined at a slower rate than those on dialysis (32).

**Figure 4:**
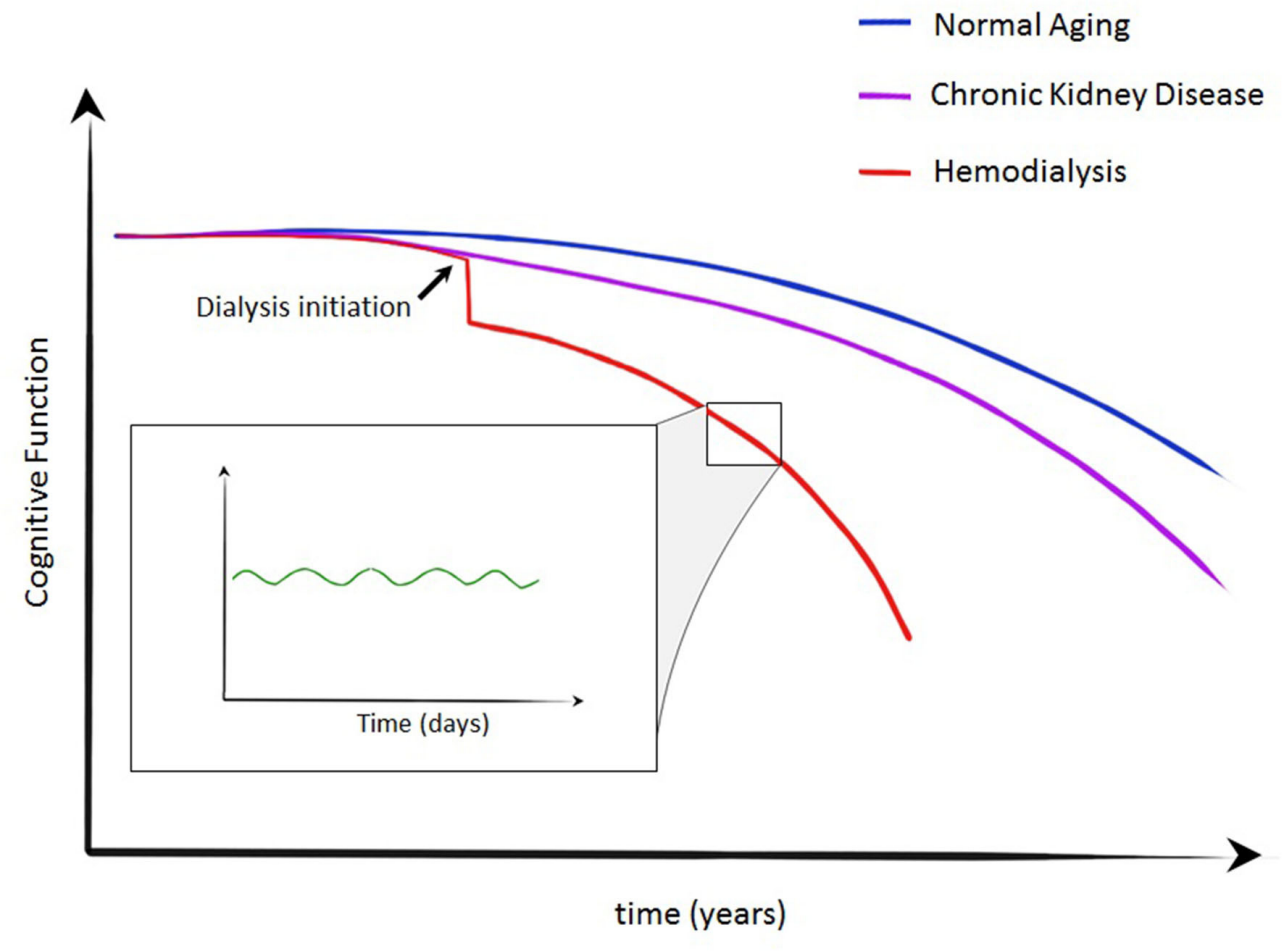
Crude conceptualization of cognitive decline in normal aging, CKD and kidney failure requiring dialysis. A stepwise decline is described at the initiation of dialysis with more rapid decline in cognitive function in those on dialysis. During individual dialysis treatments, transient cognitive decline has been demonstrated—an insult likely to contribute to the trajectory of those on dialysis.

Increasing interest is being paid to the changes in cognitive function associated with the dialysis session itself, which has consequences for determining the optimal timing for undertaking cognitive assessments. Reports are somewhat contradictory. Improvements following dialysis have been reported by Williams et al. (33), Lux et al. (34), and Schneider et al. (35) who all report multi-domain cognitive function assessments immediately prior to dialysis and comparing this to cognitive function ~24 h later. They demonstrate an improvement in multiple aspects of cognition, including memory, language, and executive function. However, one of the more revealing studies of the temporal relationship of dialysis and cognitive function was reported by Murray et al. (36). In this, they performed multi-domain cognitive assessment at multiple time points related to the dialysis treatment. Specifically, they tested 28 patients immediately before, 1 h into dialysis, 1 h after dialysis and ~24 h later. With this method they demonstrated a significant intradialytic decline in all domains of cognitive function, demonstrating the highest scores immediately before or at 24 h after dialysis (36). Therefore, one conceptualization of the progression of cognitive dysfunction in those with kidney failure could be summarized as per Figure 4. In normal aging cognitive function will decline over years, with more rapid progression in those with kidney disease. Initiation of dialysis provides an acute decline in cognitive function with more rapid decline that may be explained by repeated dialysis, capably of inducing transient cognitive decline. However, a recent prospectively randomized study designed to circumvent the learning effect of neuropsychological testing found no difference in performance during the first or second half of dialysis compared with the day after dialysis (37). The small and relatively younger and less hypertensive hemodialysis cohort studied may have confounded results; however we acknowledge this further illustrates that the effect of the hemodialysis therapy on cognition is still not fully established.

As one ages, the brain undergoes several structural changes which may have an effect on cognitive function. Atrophy is the most common change. Rate of volume loss is usually 0.5% per year after the age of 40, but with considerable variation (38). Radiological evidence of cerebral atrophy can be considered normal aging after about age 50 (39). In disease states these changes can accelerate and lead to greater loss of function.

In people requiring KRT data have shown an association with greater prevalence of atrophy (40) and onset earlier in life than the general population—reported as occurring almost a decade earlier than would be expected (41). In KFRT, cerebral atrophy is associated with cognitive impairment, duration of dialysis (42), intradialytic hypotension (43), and cerebrovascular disease (44).

White matter hyperintensities, commonly believed to represent small vessel disease are twice as common in the dialysis population compared to the general population, with a prevalence of 52 vs. 22.4% in a cohort with mean age 55.9 years (45). Advanced MR imaging is capable of assessing white matter tract integrity, prior to the onset of white matter hyperintensities. Using diffusion tensor imaging an estimation of location, orientation and anisotropy (crudely, the “direction of flow” of water molecules within white matter tracts) is of particular interest and may signify areas at risk of vascular damage. In hemodialysis patients, deterioration in markers of diffusion imply a loss of tract integrity (46), and is associated with cardiovascular instability during dialysis (47). This effect is mitigated by cooled dialysis (48), and renal transplantation (49, 50), and discussed later.

Therefore, in kidney failure requiring KRT cognitive impairment is common. Specifically, compared to age matched controls from the general population, severe cognitive impairment is more than three times more prevalent (17). It is poorly recognized, can accelerate with initiation of dialysis and may vary around the dialysis cycle. The urgent need to untangle the mechanisms of cognitive impairment in KFRT has gained increasing attention over the last two decades.

## Vasculopathy: The New Encephalopathy?—Mechanisms of Cognitive Impairment in Kidney Failure

**Figure 5:**
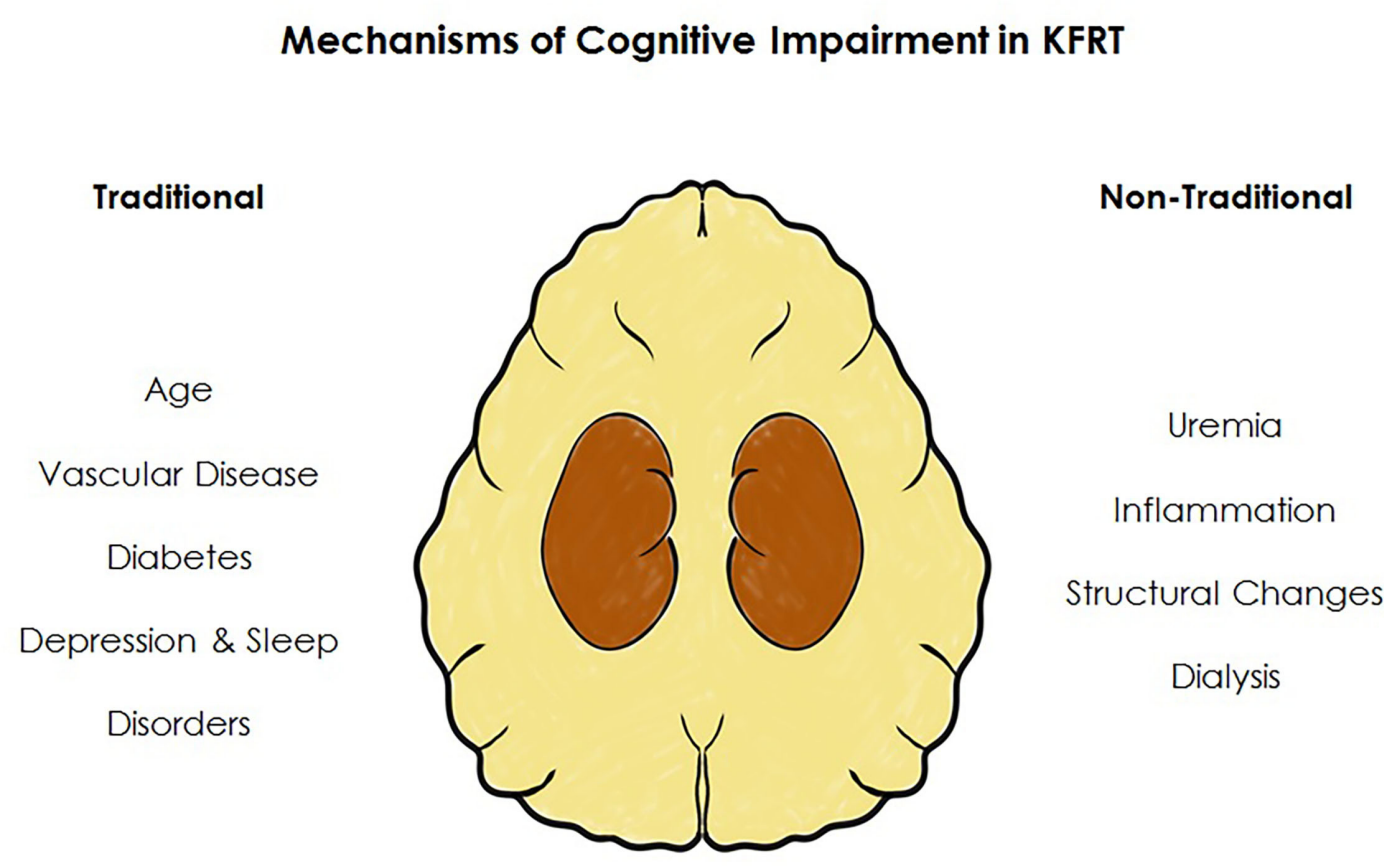
Factors associated with cognitive impairment in kidney failure. In addition to an increased burden of risk factors traditionally associated with cognitive impairment, those receiving KRT have additional factors unique to KFRT.

In this review we focus on cognitive impairment in those with kidney failure requiring KRT. However, to understand the multiple mechanisms responsible for cognitive impairment one must recognize that patients have progressed through stages of CKD accruing morbidity and complications which leads them to this point, where cognitive impairment is so highly prevalent. Once KRT is required, the process of dialysis brings unique insults implicated in the development of cognitive impairment. Although much evidence supports a vascular driven etiology of cognitive impairment in advanced CKD the process is likely multifactorial. We discuss the mechanisms of cognitive impairment unique to those requiring KRT by broadly dividing each into traditional risk factors—factors associated with cognitive decline in the general population—and non-traditional risk factors—those unique to the physiological stressors of KFRT, Figure 5.

### Traditional Risk Factors for Cognitive Decline

#### Age

Age is the single biggest risk factor for cognitive impairment in the general population (20, 51, 52). The prevalence of cognitive impairment increases with age; approximately doubling for each decade over the age of 60 years; with an estimated prevalence of 6.7% from age 60, 10.1% from 70 and 25.2% from, age 80 onwards (21) in the general population. Age is also a recognized association for cognitive impairment in the dialysis population (32). With an aging dialysis population the prevalence of cognitive impairment is high and likely to rise. However, it is worth noting in KFRT the prevalence is notably high even at a relatively young age—reaching ~10% in those aged 21–39 years (29). The rate of cognitive decline with age is highly variable between individuals. However, when comparing those on dialysis to a matched cohort of patients with CKD (GFR <30 mL/min/1.73^2^) not only is cognitive function lower at baseline in those on dialysis, but cognitive function declines more rapidly over a period of 2 years (53).

#### Cerebrovascular Disease

Cerebrovascular disease is a driving force capable of producing mild cognitive impairment and vascular dementia—the second most common cause of dementia in the general population after Alzheimer's disease (54). Compared to the general population, cerebrovascular disease is 10 times more common in those on KRT (55). Although cognitive impairment may complicate a presentation of stroke, a more insidious onset can occur through development of small vessel disease or silent infarction (56). Compared to age-matched controls those on dialysis are five times more likely to have silent cerebrovascular disease than the general population (57). In contrast to Alzheimer's disease which—in its early stages—presents predominantly with memory loss, vascular cognitive impairment classically presents with progressive loss in attentional processes and executive function (58).

As stated, the burden of cerebrovascular disease in kidney failure has led many authors to believe cerebrovascular disease plays a pivotal role in the etiology of KFRT-related cognitive impairment. Multiple supporting factors consolidate this theory. Primarily, similar to those with vascular dementia the finding of reduced executive function is the most frequently reported cognitive impairment in dialysis patients (59–61). Further, in the stages preceding KRT higher levels of albuminuria—a recognized cardiovascular risk marker—are associated with worse executive function and white matter hyperintensities on MRI (62, 63). Finally, administering more intensive dialysis—thereby improving solute clearance—does not improve cognitive function (64).

#### Cardio-Metabolic Disease

The presence of diabetes, hypertension, and cardiac failure are all associated with cognitive impairment (65–67) and all found in abundance in those with renal disease. To put this in context, data from the US and European renal registries demonstrate that the most common cause of need for KRT is diabetic kidney disease (68, 69). Further, the prevalence of hypertension in the dialysis population is significantly higher than the general population (70)—a factor often commented on when age and sex matched cohorts are compared. The relationship between cardiac and renal disease is firmly established, with cardiac disease listed as the most common cause of death in people needing KRT (71–73). A recent systematic review has summarized the magnitude of cognitive impairment in those with cardiac failure—with a prevalence of 43% (74). Although also assumed to be vascular, the pathophysiology of this remains elusive.

#### Depression

Cognitive impairment is a diagnostic criterion of major depressive disorder (18). However, even following treatment for the mood disorder, cognitive impairment can persist (75, 76). Usually presenting as self-reported memory loss, and often notably lacking in participant effort on testing, depression can present with marked memory disturbance a condition previously called “pseudodementia” (77).

Depression is common in KFRT and poorly recognized (78). Reported world-wide prevalence of depression in people requiring KRT is 13.1–76.3%, considerably higher than the estimate for the world's population at 3.38%, from a 2020 World Health Organization report (79). Diagnosing depression is difficult due to the overlap of symptoms brought by kidney disease such as anorexia, sleep disturbance, fatigue, and psychomotor retardation. Therefore, greater focus should be given to symptoms such as dysphoria, anhedonia, worthlessness/guilt, suicidal ideation, and cognitive dysfunction. Although increasingly recognized as clinically important, outside of research, regular screening for depression is not considered routine in the clinical care of those with kidney failure (80). Depression is associated with a wide range of cognitive impairment including impaired attention, decision making, memory and social cognition (81). The combined use of both cognitive behavioral therapy (CBT) and the SSRI sertraline are recommended in treating symptoms of depression—extrapolated from evidence from in the general population (82). There are data to support use of CBT in kidney failure (83) but attempts to identify a beneficial antidepressant to improve mood are yet to be conclusive (84, 85). Further, it remains unclear if cognitive function would be improved in people requiring KRT.

#### Sleep Disorders

The need for sleep varies significantly between individuals, with the average sleep length of 7–8.5 per day (86). The effect of sleep deprivation is all too familiar in medicine. Acutely, those at the end of a night shift may have been aware of altered cognitive function and all are likely to have witnessed patients with delirium worsened by sleep deprivation. Sleep deprivation can be classified as acute or chronic and partial or complete. In the general population sleep deprivation is associated with a number of cognitive deficits including attention, working memory, long-term memory executive function, and decision making (86). Recovery from sleep deprivation is possible if normal sleep regulation can resume. However, the recovery from chronic deprivation can take longer (87). Therefore, those with chronic partial sleep deprivation due to underlying chronic illnesses are unlikely to experience recovery unless provided with a treatment that results in significant alleviation of disease burden.

Sleep disturbances including insomnia, fragmented sleep or reduction in total sleep time are common in people on KRT (88). The prevalence is high, estimated at 44–95% of patients (89). Compared to age and sex matched healthy controls sleep quality is worse in those on dialysis (90). The underlying mechanisms for poor sleep are unclear. Likely hypotheses include physical factors such as daytime somnolence, sleep apnea, restless legs syndrome (91) and individual patient experience (92) such as worry and dialysis related disruptions. Alterations in brain neurochemistry are also thought to contribute. In animal models of CKD, serotonergic neurons which influence sleep-wake patterns and memory show increased activity (93). Serotonergic neurons are also recognized to contribute to depression and anorexia in CKD. The actions of neurotransmitters in kidney disease remain an area of great interest and complicated by such functional overlap. This may partly explain why observational studies have revealed association between sleep deprivation and cognitive impairment in the first year of dialysis (94) and depression in those on maintenance dialysis (95). The mechanisms underlying sleep and neurodegeneration is an active area of research and the most promising explanations center around the glymphatic system and clearance of potential toxins while sleeping (96). The detailed interaction of kidney disease and neurochemistry is beyond the scope of this review and well described elsewhere (93). Thus, a potential synergy between kidney disease and the consequences of poor sleep seems biologically plausible.

### Non-traditional Risk Factors for Cognitive Decline

#### Uremic Toxins

Advanced CKD is associated with a high burden of toxic metabolites, many of which are not routinely measured in clinical practice. In the list of uremic solutes held by the European Uraemic Toxins Work Group several distinct “uremic toxins” are recognized to have a neurological or central nervous system pathological effects (97). As described earlier, the process of dialysis can improve cognitive dysfunction in the acute setting in those with high levels of uremia. However, the persistence of cognitive impairment following dialysis initiation, and the failure of increased dialysis clearance to improve cognition (64) implies if cognitive dysfunction is toxin driven, then such neurotoxic substances are ineffectively or incompletely removed by the process of dialysis.

In health an intact blood brain barrier may provide protection, however this barrier is dysfunctional in advanced CKD (98). Particular interest has been given to neuropeptide-Y—implicated in neurodegenerative conditions such as Alzheimer's and found in higher concentrations in those with cardiovascular disease and advanced CKD—and markers traditionally associated with mineral bone disease in CKD—Klotho and FGF-23(93). Higher serum concentrations of neuroactive mediators capable of crossing the blood-brain barrier can lead to cerebral osmotic dysregulation. Recent data have demonstrated a significant fall in such neurochemical concentrations following transplantation (49).

#### Inflammation

Inflammation has been implicated in studies of neurovascular dysfunction (99). The resultant endothelial dysfunction can promote vascular leakage, protein extravasation and, as mentioned, contribute toward blood-brain barrier dysfunction.

CKD (100) and the process of dialysis are pro-inflammatory states (101) associated with a high cardiovascular disease burden. In an observational study higher levels of hsCRP, fibrinogen and IL-1b were associated with cognitive impairment (102). There are no studies which support treating inflammation to improve cognitive function.

#### Dialysis Modality

More recent developments have pointed toward an association with the process of dialysis itself. Dialysis is capable of altering cerebral blood flow which is associated with altered cognitive function (103). Polinder-Bos et al. demonstrated with PET-CT imaging an intradialytic decline in cerebral blood flow, associated with ultrafiltration rate and volume, temperature and pH (104). The hypothesis was that recurrent intradialytic decline in cerebral blood flow could predispose to cerebral ischemic injury. In 2019, our group demonstrated real-time intradialytic decreases in cerebral arterial flow correlating with worsening cognitive function (103). Receiving a kidney transplant produced an improvement in cognitive function and had a positive effect on markers of cerebral diffusion.

Kidney transplantation is clearly the gold standard, consistently improving neurochemistry, cerebral blood flow, white tract integrity and cognitive function. However, where dialysis is necessary, the use of peritoneal dialysis appears “protective” to cognitive function (105). In a well-designed study Neumann et al. explored the effect of peritoneal dialysis over hemodialysis on cognitive function. One must appreciate that by nature of the treatments, patients who opt for one form of dialysis or the other are inherently different, since peritoneal dialysis requires greater patient engagement and education to succeed. Therefore, in an attempt to overcome this, propensity score matching was utilized by Neumann et al. to create matched cohorts of hemodialysis and peritoneal dialysis treated patients with KFRT. Matching for age, education level, employment status, and comorbidity they created two groups of ~100 participants and performed tests of executive function, attention and self-reported cognitive function at baseline and 12 months. Although in this study both groups demonstrated an improvement in cognitive function at 1 year—the improvement was more marked in those on peritoneal dialysis (105).

As shown, the mechanisms underpinning cognitive impairment in kidney failure with replacement therapy are complex and multifactorial. Vascular burden, cerebral alterations, and the recurrent vascular insult from dialysis all have a plausible role. Exploring modifiable factors is essential to developing strategies to reverse or limit the burden of cognitive impairment. However, before the introduction of new treatments or alteration to existing, one must ask—what are the implications of cognitive impairment and why does this matter?

## “Dialysis Blurs The Mind” –Patient Implications of Cognitive Dysfunction in Patients Requiring Dialysis

As previously mentioned, cognitive impairment in people requiring KRT is under-recognized by physicians. An understanding of the experiences of our patients and the “real-life” implications of cognitive dysfunction is essential to improve assessment and patient-centered care. “Brain fog” is a recognized experience amongst patients on dialysis and a hot topic of conversation on patient-led forums (106). Self-reported scores of concentration and memory impairment in KFRT patient populations are associated with measures on objective executive function testing (107). One study examined stage 5 CKD patients' self-recognition of concentrating ability and memory and found it was significantly associated with their dialysis modality choice (107). The conscious perception of an individual to their cognitive decline may have a negative impact on their self-efficacy and subsequent engagement with aspects of healthcare which require more functional independence.

Impaired health literacy is associated with cognitive decline (107). There is an increasing emphasis on the facilitation of patient-led decision making and self-management for long-term conditions with associated better patient outcomes. This shift from physician-driven paternalistic models of care clearly has the potential to be undermined by the burden of multi-domain cognitive impairment in patients with KFRT if not supported adequately. The relationship between cognition, affective disorders and complex decision making has been relatively under-studied in KFRT (107). This is despite the cognitive demands of abstract KRT planning decisions and, once established on dialysis or even having undergone transplantation, the cognitive adaptability that is necessary to engage with a dynamic chronic health condition and the unavoidable healthcare activity burden associated with it. Cognitive impairment has been identified as a key factor contributing to decisions to withdraw from dialysis therapy (108). Ensuring patients with cognitive impairment can exert their autonomy and engage in complex advance care planning decisions is clearly challenging.

Cognitive impairment in people requiring KRT has been associated with a decreased quality of life (109). The psychological impact of “machine dependency” in hemodialysis treatment has been well-documented and inadequate education and preparation for dialysis contributes to the associated psychological stress. Cognitive impairment may impact on successful kidney replacement therapy education (110). Furthermore, as previously discussed, affective disorders are more prevalent in patients with cognitive impairment, and depression is associated with poor outcomes in hemodialysis patients in terms of lower quality of life, non-adherence to treatments, increased requirement for healthcare intervention and increased mortality (110, 111), Despite the vulnerability of this population, the response of hemodialysis patients with concurrent depression and cognitive impairment to pharmacological treatment for affective symptoms has not been well-evaluated (111).

Cognitive impairment has been associated with significantly higher mortality in the hemodialysis population (109). One study suggested the higher mortality is related to the impact of executive dysfunction on the complex decision making required to engage with chronic disease management (112). The management of kidney failure carries a significant burden for patients in terms of polypharmacy, restrictive lifestyle choices related to diet and fluid intake, and time spent in hospital settings. Hemodialysis patients with cognitive impairment have an increased risk of hospitalization, increased length of hospital stay and utilize a greater number of healthcare resources, including dedicated healthcare staff time (109, 113). Cognitive impairment can manifest as disruptive behavior and non-concordance with treatments (109, 110). Medication non-adherence in hemodialysis populations has been estimated as high as 58.2% in those with cognitive impairment, in comparison to 25% in the non-cognitively impaired general population (110, 111). Adherence to treatment can be taken into consideration during assessment for suitability of future kidney transplantation therefore this has potentially significant implications.

Considering the implications for patients on KRT with cognitive impairment beyond the healthcare setting, a US based study found independent correlation with self-reported functional dependence in activities of daily living with cognitive impairment, and specifically with impairment in the domain of executive functioning. Functional dependence was noted in 70% of patients with cognitive impairment in this study (114). The mechanisms driving this association are speculative, including the shared relationship cognitive impairment and functional dependence have with frailty and cerebrovascular disease. The impact of cognitive decline in engaging with social interactions and physical exercise which are protective factors for functional independence likely contributes (114). Furthermore, there is a small amount of data that suggests the cohort of patients with cognitive impairment in context of CKD is at significant risk of unsafe driving. Inability to drive would have a significant impact on functional independence. There is a paucity of research, and guidelines for patients and health professionals are vague despite the potential implications for patient safety (115).

**Figure 6:**
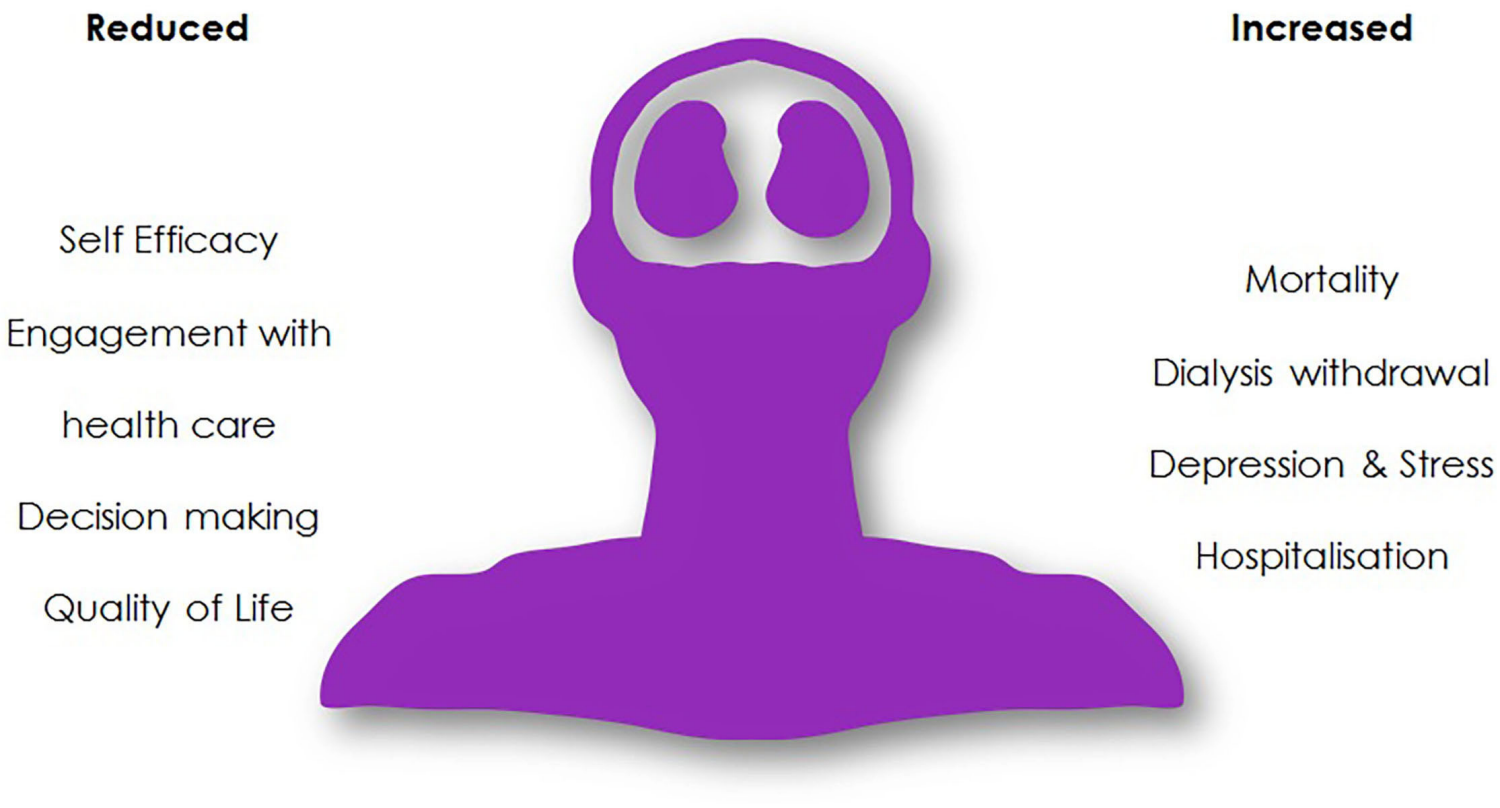
Patient implications of cognitive impairment.

Cognitive impairment in the context of KRT clearly has significant implications for patients and their healthcare providers, Figure 6. Healthcare rationing decisions on the basis of cognitive impairment have been previously highlighted given the expense associated with KRT and the perceived benefits to a cognitively impaired population (113). Identifying individuals with cognitive impairment is important to enable appropriate adaptations to their care to be made. Strategies for reducing the progression of cognitive impairment in this cohort are clearly welcomed.

## Determining Dysfunction—Cognitive Assessment in Clinical Practice

When considering cognitive problems in people living with renal disease, a common question is “what is the best cognitive test?” Unfortunately, there is no simple answer to this question, any more so than there is a simple answer to the question “what is the best method of assessing for kidney disease?” The optimal approach to assessment is dependent on multiple factors, some related to the test and some related to the context of the testing. Here, we offer practical advice and clinical applications that should allow for a more informed approach to cognitive testing within a renal service.

An important first step is to be clear on the purpose of testing. Cognitive testing can operate at many different levels, from very brief tools that can be performed at scale and offer a form of cognitive “triage,” to neuropsychological batteries that detail function across the many differing cognitive domains but that are probably only appropriate for specialist assessment services. In Table 1 are listed potential cognitive test scenarios, suitable tests and some renal medicine “equivalents.”

**Table 1:** Cognitive testing in advanced chronic kidney disease.

**Cognitive test scenario**	**Considerations**	**Potential cognitive tests**	**Renal testing “equivalents”**
Informal assessment	• Usual practice • Poor accuracy (insensitive and non-specific)	Judging cognition based on clinic consultation	Palpation for pedal oedema
Cognitive Triage	• Very brief (<5 mins) • Acceptable • Minimal training • Blunt assessment	• Single screening questions • Four point AMT • Mini-Cog	Dipstick urinalysis
Multi-domain screen	• Brief (<20 mins) • Training required • Global assessment only	• MMSE • MoCA • RUDAS	Laboratory urea and electrolytes
Multi-domain assessment	• Often >60 mins • Needs specialist expertise • Detailed assessment	Neuropsychological battery	Renal biopsy
Diagnostic formulation	• Clinically relevant • Multiple consultations • Needs specialist expertise and ancillary tests	Multidisciplinary clincial diagnosis of dementia	Multidisciplinary clinico-pathological diagnosis of glomerulonephritis

*MMSE, Mini-Mental State Exam; MoCA, Montreal Cognitive Assessment; RUDAS, Rowland Universal Dementia Assessment Scale*.

In clinical practice, testing that operates at the triage or screening level is likely to be the most pragmatic. Even here the choice of test is not straightforward, with a vast array of cognitive screening tests available and frequent new tests described in the literature (116). Certain practical considerations can help “narrow the field.” Does your service have the approvals or finance to use a copyrighted test? Traditionally popular tools such as Folstein's Mini Mental State Examination (MMSE) (117) now have copyright enforced and as a result many are looking for free to access alternatives. Does the test require training and is the training freely available? The introduction of mandatory training for the Montreal Cognitive Assessment (MoCA) (118) will raise standards and consistency in the use of the test, but the cost of the training has lessened the appeal of the test for many. The population to be tested also needs some thought, for example, are test versions available that are language and culture appropriate? The Rowlands Universal Dementia Assessment Scale (RUDAS) is a screening tool specifically designed to minimize educational and cultural biases.

Finally, the properties of the test itself should be considered. The classical test metrics of validity, reliability and responsiveness are detailed in academic texts, but often it is other aspects of the test that determine the practical clinical utility such as the feasibility of applying the test within a given clinical setting or the acceptability of the test to the patient and the person performing the testing. A thirty-minute global cognitive screen may have perfect psychometric properties but simply not be possible to administer within the constraints of an over-booked outpatient clinic or in a busy emergency department. The existing requirement for long and frequent healthcare attendances for patients on hemodialysis may limit additional attendance out with hemodialysis sessions for cognitive assessments. The common co-morbidities that occur in patients with renal disease may have impairments that complicate testing. For example, the visual impairment from diabetic eye disease, or aphasia from a stroke will all necessitate an adapted approach. As previously mentioned, there is concern regarding the systematic bias introduced into cognitive impairment epidemiology literature in KFRT populations due to the unsuitability of selected cognitive tests for those with physical disability from cerebrovascular disease and peripheral vascular disease.

If after all these considerations there is a still a choice between differing test options, then the accuracy of the test can help decide the preferred tool. When considered as a singular concept, the accuracy metrics of common screening tools are often fairly similar (117, 118). However, test accuracy is comprised of the two complementary measures of sensitivity and specificity and the relative balance of these two will differ between tests (119). Here again, the rationale for the test needs to be considered. If the intention is to capture everyone with potential cognitive issues, even at the cost of erroneously labeling people as having cognitive impairment (false positives) then sensitivity should be preferred. If the intention is only to select those who truly have cognitive issues, even at the risk of missing some people with impairments (false negatives) then specificity should be preferred. For initial cognitive screening, sensitivity tends to be favored, as all those who screen “positive” should have further assessment that discriminates true cases from false positives. Although there are no guidelines stating the optimal screening test to identify cognitive impairment or dementia in the KFRT population, some validation studies have identified that the Montreal Cognitive Assessment (MoCA) tool is one of the more sensitive for detecting executive dysfunction and thus cognitive impairment in the KFRT population (23, 30). The importance that people living with renal disease ascribe to cognitive testing and potential false positive and false negative scenarios is an area ripe for further research.

## Improving the Outcomes—Management of Cognitive Impairment in KFRT

Evidence based treatments for cognitive impairment in patients with kidney failure have proven elusive to date. There are a number of challenges to overcome in establishing therapies for cognitive impairment in this population. First, as cognitive impairment is multifactorial in etiology a single agent is unlikely to address the multiple pathological mechanisms involved. Establishing the optimal assessment to use as the end point in clinical trial of possible interventions is required. Some trials use radiological changes on brain imaging (48), whilst others use cognitive assessment tools such as MoCA (120). As multiple observational studies have demonstrated correlations between cerebral perfusion and either radiological changes associated with cognitive impairment in the population or makers of cognitive performance (103, 104, 121), it may be reasonable to use a measure of cerebral perfusion as a surrogate end point in early phase clinical trials of an intervention for cognitive impairment. However, for definitive trials a clinical endpoint would usually be required before license approval.

The randomized controlled trials that have been performed or are ongoing in this area can be categorized into three groups of interventional strategies; trials of interventions around hemodialysis treatment aimed at minimizing hemodynamic instability on hemodialysis, “lifestyle” interventions which aim to assist cognitive performance and finally “conventional” clinical trials of an investigational medicine (or supplement), aimed at improving cognition vs. placebo or no medicine.

In one of the larger randomized controlled trials (RCT) in this area, performed in 73 patients requiring hemodialysis randomized to “cool” dialysate (0.5°C below core body temperature) compared to 37°C, cool dialysate was associated with minimal changes on brain MRI on follow up, compared to progressive changes in brain composition such as increased fractional anisotropy and reduced radial diffusivity on follow up imaging over 6 months (48). The authors attribute this to better hemodynamic stability on dialysis. The implications of this intervention on cognitive function was not assessed in this trial but will be addressed in the ongoing e-CHECKED trial (122), whilst the larger MY-TEMP cluster randomized controlled trial will assess the effect of cool dialysate on hard outcomes such as survival and cardiovascular events including stroke (123).

In older adults in the general population, both exercise and cognitive training have been demonstrated to have some impact as non-pharmacological intervention to prevent cognitive decline (124, 125). In this setting, it appears that exercise training protects executive function (126). Moreover, exercise training can also be delivered during dialysis, often using cycling, and has also a wide range of potential benefits including greater cardiovascular and vascular health (127). Cognitive training has been shown to have beneficial impact on multiple domains of cognitive function, including executive function, memory, abstraction, and verbal reasoning in older adults (128)—although evidence of persistence of effect or translation to improvements in function is lacking. In one small RCT (20 patients requiring hemodialysis) both intradialytic pedal exercise and cognitive training, using stimulatory games on a handheld tablet pilot study were associated with less cognitive decline in psychomotor speed and executive function when compared to routine care over 3 months (120). The investigators will follow this up with a larger 2 × 2 factorial trial to assess whether which of these interventions perform best in isolation or in combination (129).

It is harder to find any high-quality clinical trials of pharmacological interventions to improve cognition in this population. One small crossover study (*n* = 39) of valerian as a supplement in hemodialysis patients showed some benefit in the MMSE after valerian therapy (130). However, the generalizability of these findings is questionable, with a very high incidence of literacy in the group studied and it is unclear if the investigators excluded patients with advanced cognitive impairment at baseline.

Observational data has shown that treatment of renal anemia with erythropoiesis stimulating agents (ESA) increases cerebral perfusion and oxygen consumption, which is likely to be associated with improved cognition (131). However, any potential benefit of over overzealous correction of renal anemia with these agents may be mitigated by an increased stroke risk with higher hemoglobin observed in placebo controlled and/or carefully performed randomized controlled trials of anemia correction in patients with chronic kidney disease (132–134). The recent PIVOTAL trial has demonstrated that proactive iron may be a more appropriate method of treating renal anemia in patients requiring hemodialysis to improve cardiovascular outcomes with no excess risk of stroke (135). However, the effect of this approach on cognition of cerebral perfusion is unknown.

Renal transplantation remains the optimal intervention for restoring quality of life and improving life expectancy in appropriately selected people with kidney failure requiring dialysis. In small observational cohort studies of either cognitive assessment or neuroimaging in patients with kidney failure who undergo kidney transplantation, several cerebral benefits of transplantation have been observed. Transplantation has been shown to normalize concentrations of neurochemicals such as choline and myoinositol as assessed by magnetic resonance spectroscopy over 12 months (49). Similarly, in another cohort study pre- and post- kidney transplantation a variety of neural networks assessed by functional MRI including dorsal attention network, the central executive network, the auditory network, and the visual network recovered post transplantation, whereas others such as the default mode network and the sensorimotor network did not recover completely at 6 months after kidney transplantation (136). Finally, in other studies neuropsychological testing scores improved following kidney transplantation (137). All the studies around effect of kidney transplantation on cognition are limited by small sample size and biased by the fact that only the “fitter” patients with kidney failure are likely to considered kidney transplant candidates and hence these observations may not be generalized to all patients with kidney failure, Table 2.

**Table 2:** Evidence, cerebral effects, and cognitive effects of treatment strategies to improve or slow cognitive decline in KFRT.

	**Evidence**	**Summary of Cerebral findings**	**Summary of cognitive effects**
**Lifestyle**			
Physical exercise (120, 138)	Randomized controlled trials of cycling, treadmill and exercise classes over 3–12 months.	Improved cerebral blood flow	Improvements in executive function, memory, delayed recall and self-reported cognitive function.
Cognitive Training (120)	Randomized trial data of intradialytic tablet-based games	Not assessed	Improvements in psychomotor speed and executive function
**Medicinal**			
Supplements (130)	Cross-over study of valerian supplementation on MMSE score and EEG findings, *n* = 39.	No changes in EEG between groups	Improved MMSE scores in those taking valerian
ESA to correct anemia (131, 139)	Observational data. Use of ESA and effect on cerebral perfusion, oxygen consumption, event related potential and cognitive attention	ESA improves cerebral perfusion and oxygen consumption. Electrophysiology parameters improved with greater Hct (mean 42.8 vs. 31.6%).	Attention improved with higher Hct. The risk of ESA use overshadows potential benefit.
**Kidney replacement therapy**			
Cooled dialysis (48)	Randomized control trial data, *n* = 73. Measured brain diffusion parameters over 1 year in those receiving dialysis 0.5°C below body temperature.	Diffusion markers stable in those with cooled dialysis.	Cognitive function not assessed. Will be assessed in the pending e-CHECKED trial (122)
Renal transplantation (49, 50, 103)	Observational data using cognitive assessment and cerebral imaging before and after transplantation.	Improved diffusion parameters, normalization of neurotransmitters and neural networks as assessed by functional MRI.	Improvements in general cognitive status, psychomotor speed, attention, memory, and abstract thinking have all been reported

*MMSE, mini-mental state examination; EEG, electro-encephalogram; ESA, erythrocyte-stimulating agent; Hct, Hematocrit; e-CHECKED, Evaluation of the Effect of Cooled Hemodialysis on Cognitive Function in Patients Suffering With End-stage Kidney Disease; MRI, magnetic resonance imaging*.

It may be that by the time patients with progressive CKD require dialysis, the consequences of accelerated cerebrovascular disease make any decline in cognition difficult to reverse. Other interventions to improve cardiovascular outcomes such as statins which have been effective in multiple populations, have failed to demonstrated benefits in this population for this reason (140). The primary focus should be preventing cognitive decline earlier in the course of CKD. Once established on KRT, the main goal is to ensure that hemodialysis does not further exacerbate cerebral injury. Consideration may be given to the dialysis modality including using peritoneal dialysis or with hemodialysis with longer (often nocturnal) hemodialysis with less intense ultrafiltration (141) or more frequent short hemodialysis to minimize hemodynamic upset associated with hemodialysis treatment.

## Discussion and Future Directions

The burden of cognitive impairment in the KRT population and the consequences for patients and the services caring for them is substantial. Yet, despite the growing research interest, much is still unknown about the epidemiology, natural history, and mechanisms driving the impaired cognition in this population. The contribution of hemodialysis therapy to the onset and acceleration of cognitive decline has only recently been partially described. Although early data is suggestive that cognitive function has the potential to be preserved through alterations to the dialysis process, there remains no robust or universally applied intervention demonstrated to be effective. However, kidney transplantation has once again demonstrated its benefits beyond correction of renal function.

Research into the epidemiology and natural history of cognitive impairment in KFRT would benefit from universally agreed methods of cognitive assessment with appropriate adjustments made to limit systematic bias from participant exclusion and drop-out. Research using such assessments should be in the context of larger multi-center longitudinal studies. Furthermore, undertaking studies with a KRT population under the age of 65 may help differentiate cognitive impairment related to kidney failure and hemodialysis, vs. the accelerated effects of aging. It is well-established that cognitive decline is multi-factorial in this population, and patients requiring KRT have often had years of progressing through CKD stages accumulating various risk factors. The duration of renal disease rather than simply the severity of CKD stage may influence cognition (93). We propose there should be greater focus in determining how to optimally predict cognitive decline, including identifying reliable disease biomarkers, and conducting mechanistic research on earlier CKD cohorts to enable trials of earlier preventative treatments prior to irreversible brain pathology.

Cognitive screening of KRT populations would likely bring to attention a significant number of patients with undetected cognitive impairment, and there needs to be further research into management options for these patients. Randomized controlled trials of potential treatments associated with hemodialysis therapy such as dialysate cooling and intra-dialytic exercise are now forthcoming in this area. These will hopefully help guide nephrologists in appropriately adjusting hemodialysis treatments. There are further potential areas for research to be considered such as impact of hemodialysis vascular access modality and optimal ultrafiltration targets on cerebral perfusion, the role of high flux vs. hemodiafiltration on medium sized solute removal, and whether proactive iron replacement or optimizing CKD-Mineral Bone Disease can optimize cognitive function. Further consideration of methods to preserve native renal function and whether this can impact on the natural history of cognitive decline should be considered. The impact of pharmacological therapy used for dementia in the general population and for affective disorders which can impact cognitive function also needs to be ascertained in the KRT population. As previously discussed, comparable study end-points for cognitive impairment require to be established—whether by cognitive domain neuropsychological testing, or radiological studies.

Improved detection and awareness of cognitive impairment in people requiring KRT will enable care planning discussions to be appropriately adjusted and likely will require significant service delivery change. Further research defining the health behavior patterns of this population and the impact of healthcare activity on engagement and influence on poor prognosis in this patient group is needed. More research that includes patient-derived outcome measures is welcomed.

## Author Contributions

KC, TQ, PM, and MF contributed to the conception and design of the review article, wrote sections of the manuscript and contributed to manuscript revision, and approved the submitted version. MF created Figures 1, 3–6. All authors contributed to the article and approved the submitted version.

## Conflict of Interest

The authors declare that the research was conducted in the absence of any commercial or financial relationships that could be construed as a potential conflict of interest.

## Publisher's Note

All claims expressed in this article are solely those of the authors and do not necessarily represent those of their affiliated organizations, or those of the publisher, the editors and the reviewers. Any product that may be evaluated in this article, or claim that may be made by its manufacturer, is not guaranteed or endorsed by the publisher.
